# Altered spontaneous brain activity in lumbar disc herniation patients: insights from an ALE meta-analysis of neuroimaging data

**DOI:** 10.3389/fnins.2024.1349512

**Published:** 2024-02-06

**Authors:** Zhiqiang Qiu, Xiangkai Zhong, Qiming Yang, Xiran Shi, Libing He, Huiling Zhou, Xiaoxue Xu

**Affiliations:** Department of Radiology, Affiliated Hospital of North Sichuan Medical College, Nanchong, China

**Keywords:** lumbar disc herniation, spontaneous brain activity, rs-fMRI, meta analysis, ALE

## Abstract

**Objective:**

To explore the characteristics of spontaneous brain activity changes in patients with lumbar disc herniation (LDH), and help reconcile the contradictory findings in the literature and enhance the understanding of LDH-related pain.

**Materials and methods:**

PubMed, Web of Science, Embase, Chinese National Knowledge Infrastructure (CNKI), SinoMed, and Wanfang databases were searched for literature that studies the changes of brain basal activity in patients with LDH using regional homogeneity (ReHo) and amplitude of low-frequency fluctuation/fraction amplitude of low-frequency fluctuation (ALFF/fALFF) analysis methods. Activation likelihood estimation (ALE) was used to perform a meta-analysis of the brain regions with spontaneous brain activity changes in LDH patients compared with healthy controls (HCs).

**Results:**

A total of 11 studies were included, including 7ALFF, 2fALFF, and 2ReHo studies, with a total of 269 LDH patients and 277 HCs. Combined with the data from the ALFF/fALFF and ReHo studies, the meta-analysis results showed that compared with HCs, LDH patients had increased spontaneous brain activity in the right middle frontal gyrus (MFG), left anterior cingulate cortex (ACC) and the right anterior lobe of the cerebellum, while they had decreased spontaneous brain activity in the left superior frontal gyrus (SFG). Meta-analysis using ALFF/fALFF data alone showed that compared with HCs, LDH patients had increased spontaneous brain activity in the right MFG and left ACC, but no decrease in spontaneous brain activity was found.

**Conclusion:**

In this paper, through the ALE Meta-analysis method, based on the data of reported rs-fMRI whole brain studies, we found that LDH patients had spontaneous brain activity changes in the right middle frontal gyrus, left anterior cingulate gyrus, right anterior cerebellar lobe and left superior frontal gyrus. However, it is still difficult to assess whether these results are specific and unique to patients with LDH. Further neuroimaging studies are needed to compare the effects of LDH and other chronic pain diseases on the spontaneous brain activity of patients. Furthermore, the lateralization results presented in our study also require further LDH-related pain side-specific grouping study to clarify this causation.

**Systematic review registration:**

PROSPERO, identifier CRD42022375513.

## 1 Introduction

Lumbar disc herniation (LDH) is a common spinal disease, mainly due to the rupture of the fibrous ring of the lumbar disc, with the nucleus pulposus protruding from the rupture site into the posterior or spinal canal, causing adjacent spinal nerve roots to be stimulated or compressed, resulting in a series of clinical symptoms such as lower back pain, numbness and pain in the lower limbs, and even difficulty in urination and defecation (Heliovaara et al., 1987). Epidemiological investigation shows that the incidence rate of LDH is about 7.62% (Kim et al., 2018) and its prevalence is highest between 30 and 50 years old (Fjeld et al., 2019). Understanding the central nervous system mechanism of LDH-related chronic pain will help develop strategies for the evaluation and treatment of LDH-related pain.

Functional Magnetic Resonance Imaging (fMRI) is a powerful neuroimaging technique that allows researchers to investigate brain function by measuring changes in blood flow (Ogawa et al., 1990). Resting-state fMRI (rs-fMRI) is a specific application of this technology, focusing on spontaneous fluctuations in blood oxygen level-dependent (BOLD) signals while the subject is at rest. This non-invasive method has proven valuable in exploring functional activities within the brain, providing insights into various neurological and psychiatric conditions (Damoiseaux et al., 2006). Regional homogeneity (ReHo) and amplitude of low-frequency fluctuation (ALFF) serve as crucial metrics in rs-fMRI analyses, contributing to a comprehensive understanding of the nuanced dynamics of regional coherence and global amplitude in resting-state brain function (Zang et al., 2007; Wu et al., 2009). Previous studies have used rs-fMRI technology to investigate the characteristics of spontaneous brain activity in LDH patients, attempting to reveal the possible central nervous mechanism of LDH-related chronic pain. However, these findings are not entirely consistent. Zhou et al. (2018) found that compared with HCs, the patients with discogenic pain showed a significant increase in ALFF in the affective system of the pain matrix (left anterior cingulate cortex (ACC), right anterior insula/frontal operculum, and bilateral orbitofrontal cortex) and information-processing regions (middle occipital/temporal gyrus). However, a study found that (Zhao, 2016), compared with HCs, patients with LDH have increased ALFF values in the right middle frontal gyrus (MFG) and right precentral gyrus, while the ALFF value in the cingulate gyrus decreased. Another study Koefman et al. (2016) found that the right superior frontal gyrus (SFG), left supramarginal cortex, and left parietal cortex exhibit significant BOLD signal enhancement. Correlation analysis also revealed a positive correlation between the average ALFF value of the frontal lobe and the severity of pain. The differences in these results may be related to different disease durations and severities, small sample sizes, and different methodologies.

The differences in these research results make the central nervous mechanism of LDH-related chronic pain remain controversial, and the research results are difficult to generalize. Therefore, it is necessary to integrate these inconsistent results through meta-analysis methods. Activation likelihood estimation (ALE) (Toro et al., 2008) is a coordinate-based meta-analysis method that has been widely used in brain function meta-analysis in the cognitive field in recent years. It conducts three-dimensional Gaussian processing and statistical testing on the activation point coordinates reported in the included literature to locate statistically significant brain regions. This analytical method can prevent atypical results from most studies from appearing in the meta-analysis results, greatly reducing the risk of false positive results.

To address the aforementioned controversies, this study employs a coordinate-based ALE method to systematically assess and integrate their research, which may help reconcile the contradictory findings in the literature and enhance the understanding of pain associated with LDH. The meta-analysis results may serve as specific brain biomarkers, facilitating the objective evaluation of interventions and predicting treatment efficacy for LDH-related pain. This is advantageous for achieving individualized precision treatments. Moreover, the study highlights the limitations of current LDH-related research and provides methodological suggestions that may benefit future work in this field.

## 2 Materials and methods

This study has been registered on PROSPERO with registration number CRD42022375513.

### 2.1 Literature search and selection

#### 2.1.1 Retrieval strategies

The literature search was performed according to the PRISMA guidelines for reporting meta-analyses and systematic reviews (Page et al., 2021). A comprehensive search of studies published up to February 1, 2023, was conducted in the PubMed, Web of Science, Embase, Chinese National Knowledge Infrastructure (CNKI), SinoMed, and Wanfang databases. We used the keywords (“fMRI” or “functional magnetic resonance imaging” or “brain activation” or “spontaneous brain activity” or “BOLD” or “resting-state”) AND (“LDH” or “lumbar disc herniation” or “low back pain”). In addition, the references of the included studies and relevant review articles were checked for additional relevant studies, and imported all retrieved articles into the EndNote X9 literature management tool for further screening.

#### 2.1.2 Selection criteria

##### 2.1.2.1 Inclusion criteria

1. Patients had been diagnosed with LDH, and were cc for more than 3 months;
2. A whole-brain study based on rs-fMRI of patients with LDH versus healthy controls (HCs) was conducted;
3. Reported stereotaxic coordinates [in the Montreal Neurological Institute [MNI] (Mazziotta et al., 1995) or Talairach (Talairach and Tournoux, 1988) atlases];
4. More than 5 participants in each study;
5. Published in English or Chinese.

##### 2.1.2.2 Exclusion criteria

1. Patients with other chronic pain disorders;
2. The data were unavailable (e.g., missing stereotaxic coordinates);
3. The data overlapped with those of another included publication;
4. Were reviews or meta-analysis;
5. Newcastle Ottawa scale (NOS) (Stang, 2010) score less than 6.

### 2.2 Quality assessment

Two researchers conducted literature screening, data extraction, and cross-checking independently. If there were any disagreements, they were discussed to determine the final result. The NOS was used to assess the quality of the included studies. The scoring criteria included three aspects: (1) selection of research subjects, including four items (0–4 points); (2) comparability of research subjects, including one item (0–2 points); (3) evaluation of clinical outcomes, including three items (0–3 points) (Stang, 2010). The total score was 9 points. If the score of the included studies was ≥6 points, they could be included in the data analysis.

### 2.3 Data analysis

This study was conducted in the MNI standard space, so we used the coordinates reported in the Talairach standard space to perform Lancaster transformation (Eickhoff et al., 2012) using the GingerALE software Version 3.0.2 (Eickhoff et al., 2009)^1^ to obtain the MNI coordinates. Then we performed 3D Gaussian processing on the activation points in the experiment, and each voxel in the brain obtained a corresponding probability P based on d (the distance between each voxel and the activation point) and σ (the noise level, which depends on the number of subjects in the experiment). By repeating this procedure for all activation points in a single experiment, we calculated the modelled activation (MA) score for each voxel in the experiment. Then, by summing the MA scores for the same coordinate voxel in all experiments included in the study, we determined the ALE score for each voxel, resulting in an ALE map. Finally, we performed statistical testing on the obtained ALE map (Eickhoff et al., 2012). In terms of the selection of statistical testing methods, this study adopted the latest recommended method of the GingerALE software Version 3.0.2: cluster-level family-wise error (FWE, *p* < 0.05), with the following threshold settings: the number of permutation tests was 5,000, the cluster formation threshold was set to *p* < 0.001, and the statistical significance threshold was set to *p* < 0.05 (Eickhoff et al., 2016). Finally, we used the Mango software^2^ to view the threshold map of the ALE results obtained from statistical testing.

### 2.4 Sensitivity analysis

The reproducibility of the research results was tested using the jackknife (Maesono, 2011) method, in which each time one study was removed, the remaining data were subjected to ALE meta-analysis. This process was repeated, and the results after removing a study were compared with the original results (Radua et al., 2012).

## 3 Results

### 3.1 Included studies and sample characteristics

**Figure 1:**
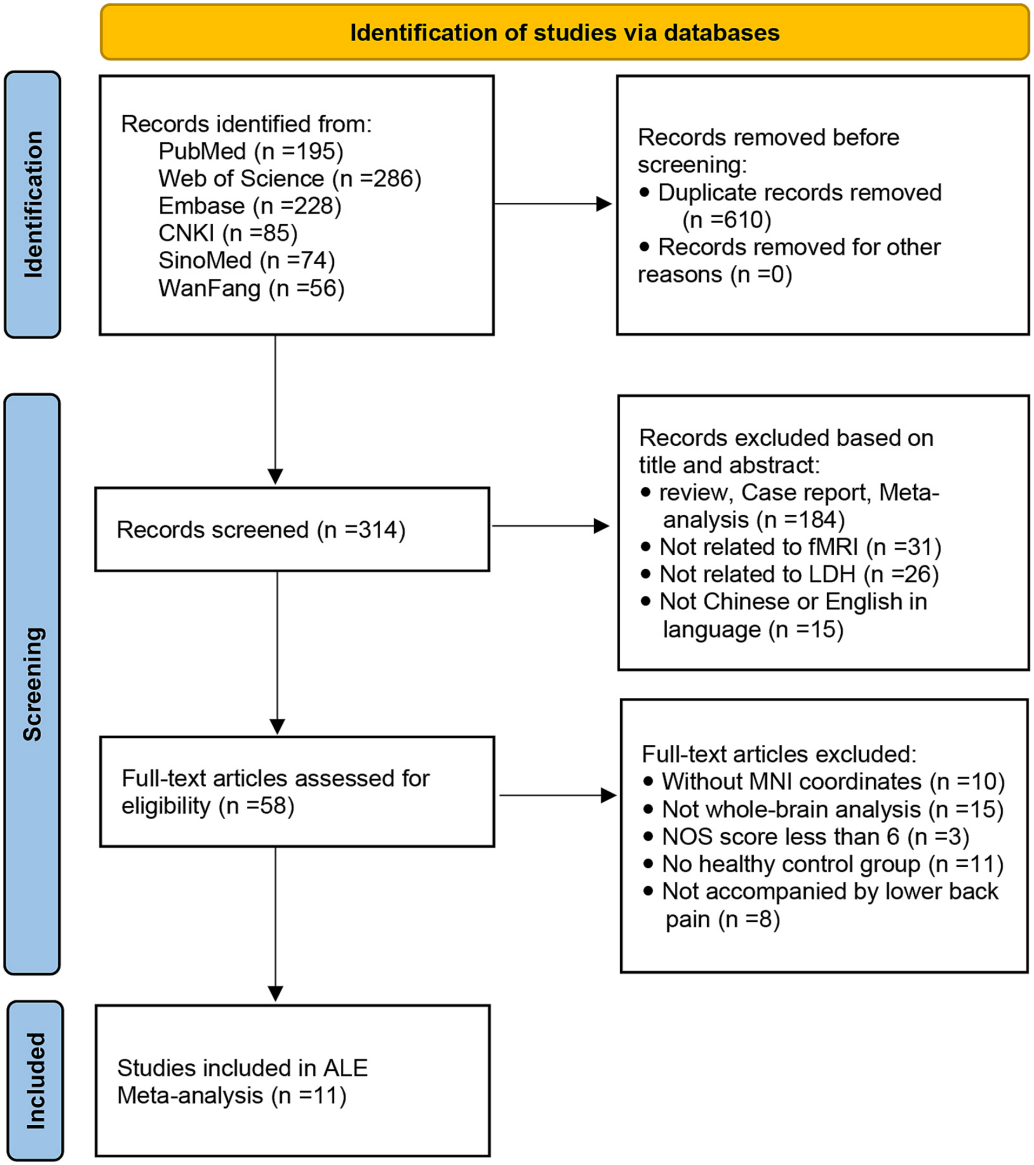
Flow chart of study selection strategy.

A flow diagram of the identification and exclusion of studies was presented in Figure 1. Following strict inclusion and exclusion criteria, this study finally included 11 studies, including 7 ALFF, 2 fALFF, and 2 ReHo studies. A total of 269 PHN patients and 277 HCs were extracted, totaling 67 differential brain regions were identified, including 44 ALFF, 3 fALFF, and 20 ReHo differential brain regions. The characteristics of the included literature are shown in Table 1. These research results were included in 2 meta-analyses: ① meta-analysis using data from ALFF/fALFF and ReHo studies; ② meta-analysis using data from ALFF/fALFF studies alone.

**Table 1:** Characteristics of included studies.

References	Sample size		Age (mean ± SD/SE)		Scanner (Tesla)	Method of analysis	Foci (n)		Standard anatomical template	Statistical threshold	Quality scores (out of 4/2/3)
	Patients	HCs	Patients	HCs			up	down			
Wen et al. (2022)	27	28	32.2 ± 9.5	31.8 ± 8.1	3 T	fALFF	2	/	MNI	p < 0.05, corrected voxel-level	4/2/1
Zhou et al. (2018)	25	27	55.16 ± 1.83	52.96 ± 1.63	3 T	ALFF	8	5	MNI	*p* < 0.01, corrected voxel-level	4/2/1
Sharma et al. (2011)	6	6	35.6 ± 2.1	38.5 ± 3.4	3 T	ALFF	5	/	Talairach	p < 0.05, uncorrected voxel-wise	4/2/1
Koefman et al. (2016)	20	20	40.35 ± 7.45	43.58 ± 8.54	3 T	ALFF	3	/	Talairach	p < 0.001, corrected voxel-level	4/2/1
Zhou et al. (2019)	25	26	55.16 ± 9.16	53.38 ± 8.34	3 T	ReHo	7	3	MNI	p < 0.01, corrected voxel-level	4/1/1
Zhu (2020)	50	47	50.92 ± 8.06	53.38 ± 8.34	3 T	ReHo	7	3	MNI	p < 0.05, corrected cluster-level	4/2/1
Zhao (2016)	12	12	47. 9 ± 6. 1	47.2 ± 6.2	3 T	ALFF	1	1	Talairach	p < 0.05, corrected cluster-level	4/2/1
Zhang (2020)	16	18	42.31 ± 10.14	42.78 ± 11.23	3 T	ALFF	4	1	MNI	p < 0.05, corrected voxel-level	4/1/1
Tan et al. (2019)	20	20	38.95 ± 11.49	38.45 ± 11.24	3 T	ALFF	2	1	MNI	p < 0.05, uncorrected cluster-level	4/1/1
Gao (2021)	55	60	48.75 ± 8.62	46.10 ± 7.79	3 T	fALFF	1	/	MNI	p < 0.001, corrected voxel-level	4/1/1
Zhang (2016)	13	13	54.23 ± 14.26	54.00 ± 14.12	3 T	ALFF	6	7	MNI	p < 0.05, uncorrected cluster-level	4/1/1

HCs, healthy controls; ALFF, amplitude of low-frequency fluctuations; fALFF, fraction amplitude of low-frequency fluctuation; ReHo, regional homogeneity; MNI, Montreal Neurological Institute.

### 3.2 ALE results

**Figure 2:**
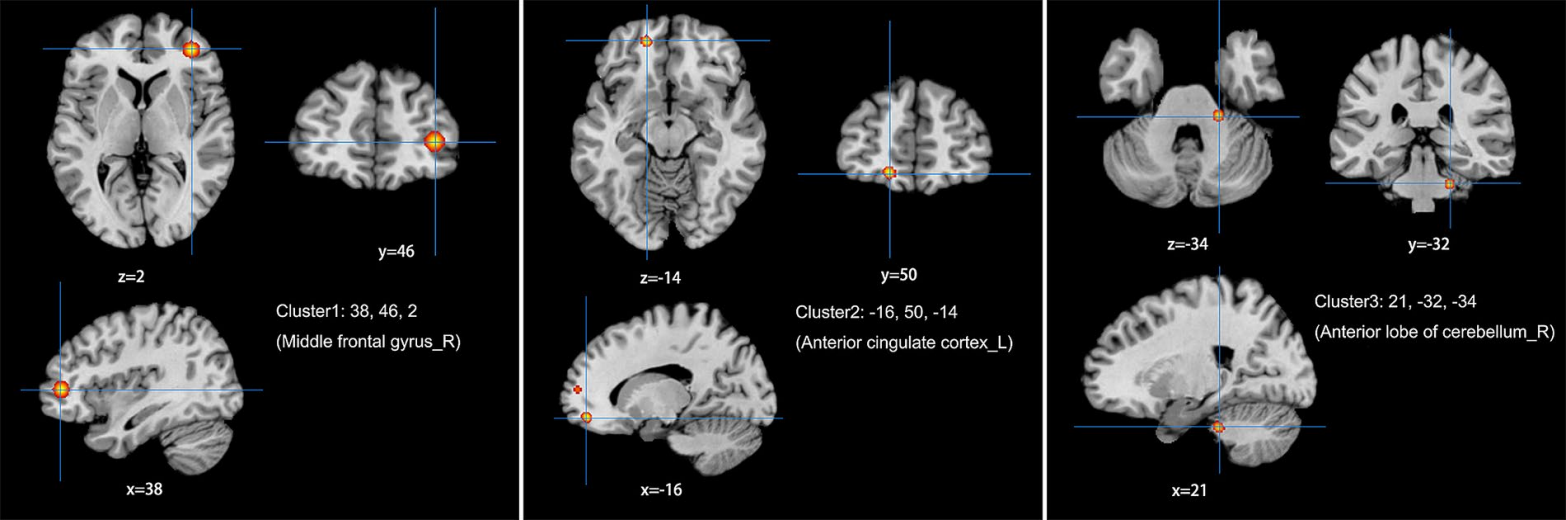
Combine ALFF/fALFF and ReHo studies to analyze the schematic diagram of brain regions with increased spontaneous brain activity in LDH patients compared with HCs. The spontaneous brain activities of the right middle frontal gyrus (MFG), the left anterior cingulate cortex (ACC) and the right anterior lobe of the cerebellum were increased. (Cluster-level FWE, *p* < 0.05, *p* < 0.001).

**Figure 3:**
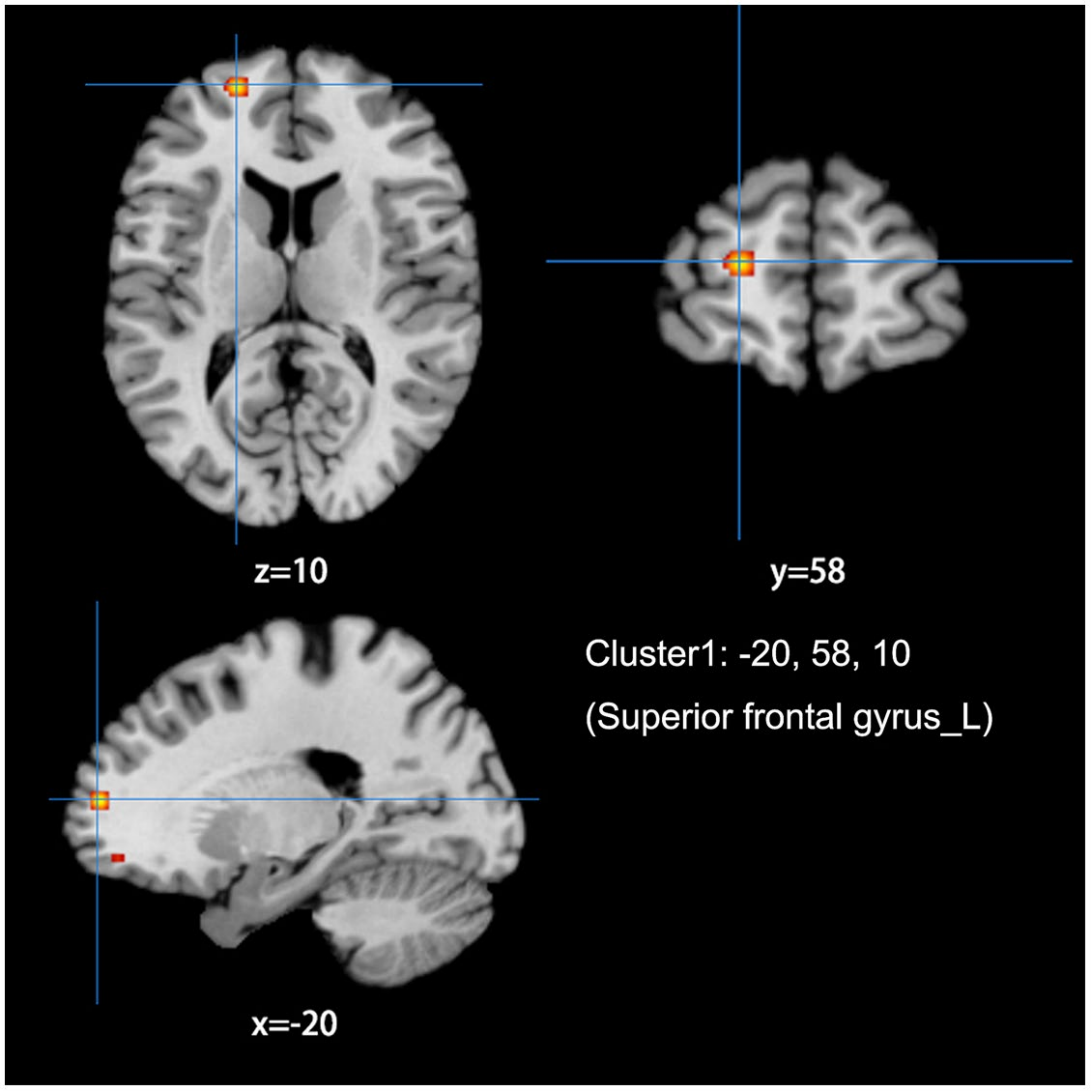
Combine ALFF/fALFF and ReHo studies to analyze the schematic diagram of brain regions with reduced spontaneous brain activity in LDH patients compared with HCs. The spontaneous brain activities of the left superior frontal gyrus (MFG) were reduced. (Cluster-level FWE, *p* < 0.05, *p* < 0.001).

**Figure 4:**
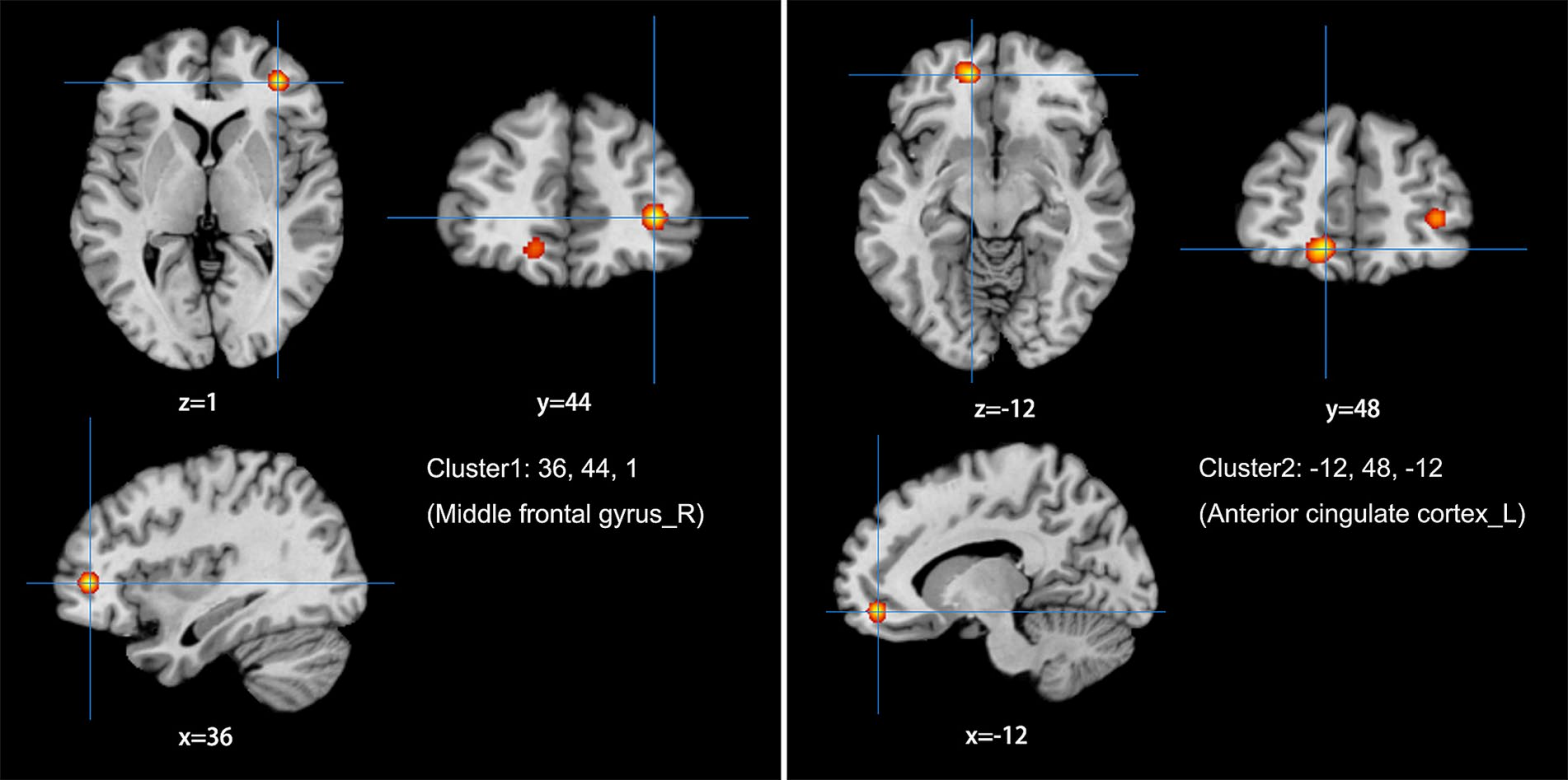
Using ALFF/fALFF studies alone to analyze the schematic diagram of brain areas with increased spontaneous brain activity in LDH patients compared with HCs. The spontaneous brain activities of the right middle frontal gyrus (MFG) and the left anterior cingulate cortex (ACC) were increased. (Cluster-level FWE, *p* < 0.05, *p* < 0.001).

The meta-analysis of data from combined ALFF/fALFF and ReHo studies showed that the spontaneous activity of the right MFG, left ACC, and the right anterior lobe of the cerebellum were increased in LDH patients relative to HCs (Table 2; Figure 2); while the spontaneous activity of the left SFG was decreased (Table 3; Figure 3). The meta-analysis of data from ALFF/fALFF studies alone showed that the spontaneous activity of the right MFG and left ACC was increased in LDH patients relative to HCs (Table 4; Figure 4), and no brain regions with decreased spontaneous activity were found.

**Table 2:** Increased spontaneous brain activity in LDH patients versus HCs: results of the meta-analysis combining ALFF/fALFF and ReHo studies.

Cluster	Side	Brain region	Peaks: MNI coordinates			Volume (mm^3^)	ALE-value	*p*-value	*Z*-value
			X	Y	Z				
1	R	Middle frontal gyrus	38	46	2	1,488	0.029	1.495	7.599
2	L	Anterior cingulate cortex	−16	50	−14	504	0.016	7.942	4.799
3	R	Anterior lobe of cerebellum	21	−32	−34	1,016	0.014	3.934	4.468

ALE, activation likelihood estimation; LDH, lumbar disc herniation; HCs, healthy controls; ALFF, amplitude of low-frequency fluctuations; fALFF, fraction amplitude of low-frequency fluctuation; ReHo, regional homogeneity; MNI, Montreal Neurological Institute.

**Table 3:** Decreased spontaneous brain activity in LDH Patients versus HCs: results of the meta-analysis combining ALFF/fALFF and ReHo studies.

Cluster	Side	Brain region	Peaks: MNI coordinates			Volume (mm3)	ALE-value	*P*-value	*Z*-value
			X	Y	Z				
**1**	L	Superior frontal gyrus	−20	58	10	480	0.015	1.784	4.635

ALE, activation likelihood estimation; LDH, lumbar disc herniation; HCs, healthy controls; ALFF, amplitude of low-frequency fluctuations; fALFF, fraction amplitude of low-frequency fluctuation; ReHo, regional homogeneity; MNI, Montreal Neurological Institute.

**Table 4:** Increased spontaneous brain activity in LDH Patients versus HCs: results of the meta-analysis from ALFF/fALFF studies alone.

Cluster	Side	Brain region	Peaks: MNI coordinates			Volume (mm^3^)	ALE-value	*p*-value	*Z*-value
			X	Y	Z				
1	R	Middle frontal gyrus	36	44	1	960	0.025	5.685	6.447
2	L	Anterior cingulate cortex	−12	48	−12	1,296	0.024	6.304	6.431

ALE, activation likelihood estimation; LDH, lumbar disc herniation; HCs, healthy controls; ALFF, amplitude of low-frequency fluctuations; fALFF, fraction amplitude of low-frequency fluctuation; MNI, Montreal Neurological Institute.

### 3.3 Quality assessment and heterogeneity analysis results

The NOS scoring results indicate that the quality scores of the included studies are generally high. However, there is still some heterogeneity among the studies, mainly reflected in: ① Studies with different test thresholds were included in this meta-analysis; ② Three studies (Sharma et al., 2011; Zhang, 2016; Tan et al., 2019) only reported results with uncorrected statistical thresholds; ③ The 11 studies included in this meta-analysis differ in terms of the participants’ ages; ④ Only two (Koefman et al., 2016; Zhu, 2020) studies reported the laterality of pain in patients with LDH.

### 3.4 Results of sensitivity analysis

Using the Jackknife method to conduct resampling on the included studies, the meta-analysis results showed that when combining the data from ALFF/fALFF and ReHo studies for meta-analysis, the repeatability of the right MFG and left ACC reached 9 times in 11 analyses, and the repeatability of the right anterior lobe of the cerebellum and left SFG reached 8 times. When using the data from ALFF/fALFF studies alone for meta-analysis, the repeatability of the right MFG and left ACC reached 7 times in 9 analyses.

## 4 Discussion

This study aims to explore consistent changes in spontaneous brain activity in LDH patients by using the ALE meta-analysis method to integrate existing rs-fMRI whole-brain studies. The results show that consistent changes in spontaneous brain activity in LDH patients may be located in the right MFG, left ACC, right anterior lobe of the cerebellum, and left SFG. In the following content, we will explore this finding and further discuss the characteristics of changes in spontaneous brain activity in LDH patients.

### 4.1 Frontal lobe

The frontal lobe is the most advanced part of brain development, consisting of the prefrontal lobe, the primary motor area before the central sulcus, and the premotor area. According to the research of Apkarian et al. (2005), the prefrontal lobe is responsible for encoding the cognitive part of acute and chronic pain, and plays an important role in evaluating pain and deciding how to respond to pain. A study Tracey and Mantyh (2007) has confirmed that the prefrontal cortex is part of the pain brain network and participates in the regulation of pain information transmission. Our meta-analysis also shows that the spontaneous brain activity in the right MFG of LDH patients is higher than that of HCs. In the 11 studies included, 6 ALFF/fALFF studies (Koefman et al., 2016; Zhang, 2016; Zhao, 2016; Zhou et al., 2018; Tan et al., 2019; Gao, 2021) and 1 ReHo study Zhu (2020) reported that the spontaneous brain activity in the frontal lobe of LDH patients increased. We speculate that the pain in LDH patients causes the increase in spontaneous brain activity in the frontal lobe, and the enhancement of frontal lobe function also plays a role in resisting pain. Koefman et al. (2016) also found a positive correlation between the average ALFF value of the frontal lobe and the degree of pain through correlation analysis, indicating that the spontaneous neural activity in the frontal lobe increases with the increase in pain. Interestingly, our meta-analysis also shows that the spontaneous brain activity in the left SFG of LDH patients decreased, and in the 11 studies included, 2 ALFF/fALFF studies (Zhang, 2016; Zhou et al., 2018) and 1 ReHo study (Zhou et al., 2019) reported this phenomenon. Zhang (2016) believed that this may be due to the long-term chronic pain, causing LDH patients to have a lack of pain cognition and emotional indifference. Dong and Zhao (2018) used voxel-based morphometry (VBM) to study chronic pain patients with LDH, and found that there was bilateral frontal lobe gray matter volume atrophy, suggesting that this long-term chronic pain may damage the frontal lobe brain structure of LDH patients. We speculate that this change might relate to the duration of LDH patients’ disease, and short-term pain might lead to increased spontaneous brain activity in the frontal lobe of patients, while with the extension of the disease, long-term chronic pain also damages the structure of the frontal lobe, resulting in decreased spontaneous brain activity in the frontal lobe of LDH patients.

### 4.2 Cingulate cortex

The cingulate cortex is an important component of the limbic system, comprising the ACC and posterior cingulate cortex (PCC). The ACC is one of the cortical representation areas of pain, and is primarily involved in encoding emotional pain information. At the same time, the ACC receives dual convergence from the medial pain pathway and limbic system pathway, and sends fiber projections to the prefrontal lobe, indicating its pivotal role in pain processing (Rolls, 2019). Our meta-analysis results also showed that the spontaneous brain activity in the left ACC of LDH patients was increased compared to that in HCs. In the 11 studies we included, 5 ALFF/fALFF studies (Sharma et al., 2011; Zhao, 2016; Zhou et al., 2018; Tan et al., 2019; Zhang, 2020) and 1 ReHo study (Zhu, 2020) reported an increase in spontaneous brain activity in the cingulate cortex of LDH patients. We speculate that LDH patients may have developed emotional disorders in long-term chronic pain, causing the cingulate cortex to compensate for encoding emotional pain information. Zhao et al. (2019) found that the functional connectivity between the cingulate cortex and bilateral MFG, precuneus, and right insula decreased; while the connectivity with the left MFG and right PCC increased, suggesting that the cingulate cortex is also closely related to the changes in the default mode network (DMN) connectivity in LDH patients. In addition, Tan et al. (2019)’s study also found that there was a positive correlation between the visual analogue scale (VAS) score and the average ALFF value of the cingulate cortex in LDH patients, and after undergoing massage therapy, the ALFF values in these regions gradually decreased and returned to normal levels, suggesting that the changes in spontaneous brain activity in LDH is reversible.

### 4.3 Cerebellum

The cerebellum is located in the lower rear part of the brain, in the posterior cranial fossa, and plays an important role in coordinating fine motor movements and maintaining body balance (Roostaei et al., 2014). Another study has shown that, after receiving pain stimuli, the cerebellum can respond through motor, cognitive, and emotional control (Moulton et al., 2010). Our meta-analysis results show that spontaneous brain activity in the right anterior lobe of the cerebellum in LDH patients is higher than that in HCs. In the 11 studies included in our analysis, 4 ALFF/fALFF studies (Sharma et al., 2011; Koefman et al., 2016; Zhou et al., 2018; Wen et al., 2022) and 1 ReHo study (Zhou et al., 2019) reported increased spontaneous brain activity in the cerebellum in LDH patients. We believe that this may be related to the decreased motor balance ability in LDH patients, which leads to compensatory increase in cerebellar activity. Given the role of the cerebellum in balance control and motor automations (Manto et al., 2012), another possible reason for this change in activity may be that patients with LDH might be constantly in a forced posture due to the LDH (i.e., trying to obtain a posture/gait that avoids pain caused by the nerve root compression). Zhang et al. (2014)’s study also showed that chronic low back pain leads to increased regional connectivity in the cerebellum during the resting state. In addition, a study Zhang (2016) has found that an increase in spontaneous neural activity in the left posterior lobe of the cerebellum is accompanied by a decrease in spontaneous neural activity in the right primary motor cortex (M1) region, indicating a coordinated role between the cerebellum and motor cortex during chronic pain. They believe that this may be related to the coordination disorder of lower limb activities in LDH patients.

### 4.4 Hemispheric lateralization outcomes of altered spontaneous brain activity in LDH-related pain

Our study demonstrates significant lateralization results (right MFG, left ACC, right anterior cerebellar lobe, left SFG). However, the extent of hemispheric asymmetry in the context of pain is not yet well understood. In experimental electric shock pain trials on healthy subjects (Symonds et al., 2006), after stimulating the left and right index fingers separately, fMRI results highlighted five brain regions (SFG, inferior parietal lobule, MFG, inferior frontal gyrus, ACC), which were highly active in the right hemisphere. A meta-analysis of 40 neuroimaging studies (Simons et al., 2014) found that in acute pain, the right amygdala is always more active, whereas in chronic pain, the left amygdala is more active. These results seem to indicate that even if pain itself is not lateralized, the frontal processing of pain may be lateralized. However, Coghill et al. (2001) discovered that in pain-free human participants, thermal pain stimulation to the left and right ventral forearms indicated contralateral activation in the primary (S1) and secondary (S2) somatosensory cortices, insular cortex, bilateral regions of the cerebellum, putamen, thalamus, ACC, and frontal operculum. In another study Barroso et al. (2020) involving osteoarthritis patients, compared to control subjects, the contralateral ACC and motor cortex showed reduced gray matter volume. So, does the lateralization results in our study also relate to the lateralization of pain caused by LDH (for instance, herniation compresses the nerve root only on one side)? Regrettably, in the studies we included, only two reported the lateralization of patients’ pain (Koefman et al., 2016; Zhu, 2020), and none conducted a side-specific grouping study. Therefore, to clarify this issue, it is necessary to conduct lateralization studies on LDH-related pain in future research.

### 4.5 Impact of activation point distribution in ALE meta-analysis

In ALE meta-analysis, a probability distribution algorithm, the more concentrated the reported coordinates of the activation points are, the greater the probability of meeting the statistical test threshold, and the more likely it is to detect possible regions of spontaneous brain activity changes (Manto et al., 2012). When the number of activation points is too small, it may be because the activation points are too dispersed to reach the statistical test threshold, and potential regions of spontaneous brain activity changes may be missed (Müller et al., 2018). This may also be one of the reasons why we failed to detect regions of spontaneous brain activity differences when only using the data (2 studies, a total of 20 activation points) from the ReHo study exclusively for meta-analysis. We have therefore abandoned the somewhat unreasonable grouping that only considered ReHo studies. Therefore, to obtain more accurate characteristics of spontaneous brain activity changes in LDH patients, further validation from multi-center, large-sample neuroimaging studies is needed.

### 4.6 Limitations

Several limitations need to be considered in this study. Firstly, even though we adopted more stringent inclusion and exclusion criteria, there is still significant heterogeneity among the studies included in the meta-analysis. (including results with uncorrected statistical thresholds, the inclusion of studies with different testing thresholds, and age differences among the studies included). Although the application of the ALE method greatly reduces the risk of false positive results, we still need to face up to the impact of this heterogeneity on the results of the meta-analysis; We also considered controlling for this potential bias, but we believe it is more appropriate to include these studies with a certain risk of false positive results than to completely ignore them. We suggest that future LDH research should adopt a more rigorous design [using brain function research recommended test thresholds, avoiding reporting results with uncorrected statistical thresholds (Bennett et al., 2009; Lieberman and Cunningham, 2009; Woo et al., 2014)]. Furthermore, as more LDH-related brain function studies emerge, future meta-analyses can systematically analyze studies from the same age level. Secondly, in all the studies we included, there is a lack of positive control groups compared to other chronic pain patients. Therefore, it is unclear whether our research results are specific, and whether this characteristic of brain spontaneous activity change is only applicable to LDH patients. We recommend adding a positive control group in future studies to assess the specificity of these changes. Finally, the ALE method used in this study is based on peak coordinates reported in existing studies, not on original imaging data. Future research could perform meta-analyses based on brain maps to obtain more accurate analysis results.

## 5 Conclusion

In summary, using the ALE meta-analysis method, based on existing rs-fMRI whole-brain research data, this article found that the possible brain spontaneous activity changes in LDH patients are located in the right MFG, left ACC, right anterior lobe of the cerebellum, and left SFG. However, due to the lack of positive control groups of other chronic pain patients in the studies we included, it is difficult to assess whether these results are specific to and unique to LDH patients. Therefore, further neuroimaging studies are needed to compare the effects of LDH and other chronic pain diseases on the spontaneous brain activity of patients. Furthermore, the lateralization results presented in our study also require further LDH-related pain side-specific grouping study to clarify this causation.

## Data availability statement

The original contributions presented in the study are included in the article/supplementary material, further inquiries can be directed to the corresponding author.

## Author contributions

ZQ: Conceptualization, Data curation, Formal analysis, Methodology, Supervision, Validation, Writing – original draft, Writing – review & editing. XZ: Data curation, Validation, Writing – original draft, Writing – review & editing. QY: Formal analysis, Methodology, Writing – review & editing. XS: Formal analysis, Methodology, Writing – review & editing. LH: Supervision, Validation, Writing – review & editing. HZ: Supervision, Validation, Writing – review & editing. XX: Conceptualization, Funding acquisition, Project administration, Resources, Supervision, Validation, Writing – review & editing.

## Footnotes

^1^http://brainmap.org/ale

^2^http://rii.uthscsa.edu/mango/
